# Optimizing cancer care: specialization, coordination and cooperation

**DOI:** 10.1054/bjoc.1999.0868

**Published:** 1999-12-08

**Authors:** A G Renehan, S T O'Dwyer

**Affiliations:** Department of Surgery, Christie Hospital NHS Trust, Wilmslow Road, Manchester, M20 4BX, UK


*British Journal of Cancer* (2000) **82**(1), 7–8
© 2000 Cancer Research Campaign
Article no. bjoc.1999.0868

Editorial

### Optimizing cancer care: specialization, coordination and cooperation

**AG Renehan and ST O'Dwyer**

Department of Surgery, Christie Hospital NHS Trust, Wilmslow Road, Manchester M20 4BX, UK

As we move towards the next millennium, providing high quality care to all patients with cancer has become a key objective for both clinicians and politicians. How to achieve this remains the subject of intense debate, particularly on issues related to specialization and centralization. Extensive reviews of cancer specialization-related studies by Stiller (1994) and Selby et al (1996) demonstrated wide variations in outcome for many common cancers but concluded that the evidence supported a case for more specialized care. Against this background, the Chief Medical Officer assembled a working party (The Expert Advisory Group on Cancer) to evaluate cancer services in England and Wales leading to the publication of the Calman–Hine Report (1995). A central premise of the report was that such variations in cancer care were unacceptable and recommended that all patients with cancer should have access to a uniformly high quality of care wherever treated. The report separated cancers into three categories – common (e.g. colorectal, lung, breast), moderately common (e.g. gastro-oesophageal, ovarian, pancreatic) and uncommon (e.g. paediatric, testicular) – to serve as an initial framework by which to consider the provision of specialized cancer services. The beneficial role of specialization for less common cancers, such as childhood malignancies, was well established (Steller and Bunch, 1990), and a network of centres providing paediatric oncology already existed. For the commoner malignancies, affecting larger patient numbers with varying presentations, it was envisaged that cancer care be delivered through an integrated network of cancer units (mainly based in district general hospitals) and cancer centres (hospitals offering dedicated oncology, radiotherapy and specialized surgical services), in close collaboration with primary and palliative care groups.

The paper by Stockton and Davies in this issue stands at the crossroad of cancer specialization in the UK. It is a comparative study of outcome for six different cancers between patients treated at hospitals with radiotherapy and oncology services (Group 1) versus district general hospitals (Group 2). Although the study period spanned from 1989 to 1993, and hence predated the Calman–Hine era, the Group I and II hospitals are broadly equivalent to cancer centres and cancer units respectively. Hence, this study provides a baseline against which future audits may be judged. The principal finding, that adjusted survival was significantly better in Group 1 hospitals, supports the aforementioned evidence in favour of cancer specialization and, on the face of it, would appear to support the Calman–Hine philosophy. The factors giving advantage to the cancer hospitals are unclear but that of stage migration is perhaps understated. Referred to as the 'Will Rogers' phenomenon, shifts in tumour stage (mainly upstage) as a consequence of improved diagnostic technology (e.g. radiology and pathology) are a major source of misleading survival statistics in cancer (Feinstein et al, 1985). In common with others, the study also found that specialized care was most beneficial in certain cancers, namely breast, ovarian and rectal tumours. While the explanation for this is as yet unclear, it appears that these cancers are particularly sensitive to suboptimal management, and hence there is a distinct need to prioritize resources and training in these areas.

An alternative pessimistic interpretation of Stockton and Davies' study is that it raises doubts about the potential delivery of an equally high-quality care across both cancer centres and cancer units. This undermines the Calman–Hine principles and potentially introduces negative competitiveness between centres and units. Such judgement seems unjust to those dedicated to the delivery of specialized cancer services within a district hospital as there is evidence suggesting that high quality care can be successfully established where specific commitments are made. For district hospitals participating in multi-centred trials, results are comparable with specialist centres, though adherence to protocols off trials may be poor (Sengupta et al, 1999). A study from Manchester (Kingston et al, 1992), has shown that specialist colorectal cancer surgeons in district hospitals can produce similar results to their colleagues in teaching hospitals, and for rectal cancer, there is well-documented evidence (albeit non-comparative) that outcome from surgical treatment in the hands of an enthusiast is comparable to best trial results (Heald and Ryall, 1986). With surgery playing a pivotal role in the initial management of many such cancers, the emphasis must be on the quality and training of surgeons treating these patients (Chan, 1999; Renehan and O'Dwyer, 1999). Numerically, the majority of common cancers will continue to be treated in the cancer units where existing cancer specialization must be nurtured and expanded.

It seems likely that the findings of Stockton and Davies are reproducible throughout the UK, reflecting an imbalance between cancer centres and cancer units. The recommendations of Calman–Hine were designed to operate within the existing system of district, teaching and cancer hospitals in the UK, but unfortunately, the envisaged 'integrated network' has failed to materialize. Achieving the targets of *Saving Lives: Our Healthier Nation* (1999) to improve cancer survival over the next decade, is not simply about injecting manpower and finances, but about the development, nurturing and sustainment of mutually supportive and appropriately trained specialist teams. Only when clinicians, purchasing groups and ministers cooperate, will cancer survival in the UK mirror that of other developed nations.

*Correspondence to:* ST O'Dwyer

### REFERENCES

Expert Advisory Group on Cancer (1995) A Policy Framework for Commissioning Cancer Services. Guidance for Purchasers and Providers of Cancer Services. (The Calman-Hine Report). Department of Health: London

Chan KK (1999) Does the surgeon matter in the management of ovarian cancer? *Br J Cancer* **80**: 1492–1493

Feinstein AR, Sosin DM and Wells CK (1985) The Will Rogers phenomenon. Stage migration and new diagnostic techniques as a source of misleading statistics for survival in cancer. *N Engl J Med* **312**: 1604–1608

Heald RJ and Ryall RDH (1986) Recurrence and survival after total mesorectal excision for rectal cancer. *Lancet* **i**: 1479–1482

Kingston RD, Walsh S and Jeacock J (1992) Colorectal surgeons in district general hospitals produce similar survival outcomes to their teaching hospital colleagues: review of 5-year survivals in Manchester. *J R Coll Surg* **37**: 235–237

Renehan AG and O'Dwyer ST (1999) Surgeon-related factors and outcome in rectal cancer. *Ann Surg* **229**: 442–443

Secretary of State for Health (1999) *Saving Lives: Our Healthier Nation*. The Stationary Office: London. (*http://www.official-documents.co.uk/documents/cm43/4386/4386.htm*)

Selby P, Gillis C and Haward R (1996) Benefits from specialised cancer care. *Lancet* **348**: 313–318

Sengupta PS, Jayson GC, Slade RJ, Eardley A and Radford JA (1999) An audit of primary surgical treatment for women with ovarian cancer referred to a cancer centre. *Br J Cancer* **80**: 444–447

Stiller CA (1994) Centralised treatment, entry to trials and survival. *Br J Cancer* **70**: 352–362

Stiller CA and Bunch KJ (1990) Trends in survival for childhood cancer in Britain diagnoses 1971–85. *Br J Cancer* **62**: 808–815

Stockton D and Davies T (1999) Multiple cancer site comparison of adjusted survival by hospital of treatment: an East Anglian study. *Br J Cancer*

*© 2000 Cancer Research Campaign*

